# Ghost Imaging Based on Deep Learning

**DOI:** 10.1038/s41598-018-24731-2

**Published:** 2018-04-24

**Authors:** Yuchen He, Gao Wang, Guoxiang Dong, Shitao Zhu, Hui Chen, Anxue Zhang, Zhuo Xu

**Affiliations:** Electronic Materials Research Laboratory, Key Laboratory of the Ministry of Education & International Center for Dielectric Research, Xi’an Jiaotong University, Xi’an, 710049 China

## Abstract

Even though ghost imaging (GI), an unconventional imaging method, has received increased attention by researchers during the last decades, imaging speed is still not satisfactory. Once the data-acquisition method and the system parameters are determined, only the processing method has the potential to accelerate image-processing significantly. However, both the basic correlation method and the compressed sensing algorithm, which are often used for ghost imaging, have their own problems. To overcome these challenges, a novel deep learning ghost imaging method is proposed in this paper. We modified the convolutional neural network that is commonly used in deep learning to fit the characteristics of ghost imaging. This modified network can be referred to as ghost imaging convolutional neural network. Our simulations and experiments confirm that, using this new method, a target image can be obtained faster and more accurate at low sampling rate compared with conventional GI method.

## Introduction

Ghost imaging (GI) is a relatively new imaging method compared with conventional imaging methods. Since the first GI experiment conducted by Pittman and Y.H.Shih in 1995^1^, ghost imaging has made great progress and extended into many related fields^2–16^. Although GI shares a similar imaging scheme with single-pixel imaging (SPI), their studies were conducted separately in computer science and optics^17^. While GI has advantages over conventional imaging methods, its imaging speed (without substantial image quality loss) remains a big problem. Several attempts to improve the imaging speed were reported recently. Most of them focus on either improving the method of data acquisition^18–23^, or the processing method in GI^24–29^. A widely used method in ghost imaging is the basic correlation method^1^. Unfortunately, the basic correlation method requires long data acquisition times. Compressed sensing (CS) is increasingly used for GI because it permits the reconstruction of targets even at a low sampling rate^30,31^. O.S. Magana-Loaiza *et al*. proposed a proof-of-principle object-tracking protocol using a ghost imaging scheme^32^. They utilized the CS protocol to minimize both the number of photons and the number of measurements required to form a quantum image of the tracked object. Recently, Z. Yang *et al*. introduced a new technique, which allows sensing of an object with fewer measurements than to other schemes that use pixel-by-pixel imaging^33^. They achieved object identification with a technique based on CS, confirming the potential impact of CS. However, CS requires a large number of computations, causing long signal processing times that limit the imaging speed of GI.

**Figure 1:**
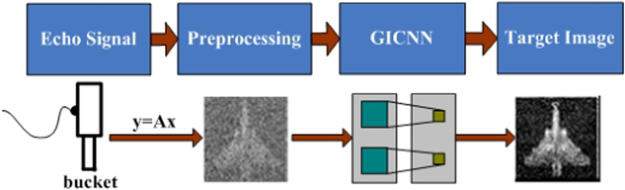
Schematic Diagram of DLGI. *y* represents the data received by the bucket detector, *A* is the speckle irradiated on the target, and *x* is the target to be reconstructed. A rudimentary image of the target can be obtained quickly using a low sampling rate with the CS algorithm. This rudimentary image is used as the input of the GICNN. The image with full sampling rate is treated as the label. After training, the target image can be obtained quickly and accurately, even if data pass through the GICNN at a low sampling rate.

In this study, we focus on methods that can quickly reconstruct an image along with high accuracy, as well as reduce the computational effort. To achieve this, we replaced the current method for GI with a method that benefits from “deep learning”. Deep learning is the most popular system optimization method and represents an extension of machine learning. It is applicable to many domains of science, business and government^34–37^. We attempt to utilize the advantages of deep learning to enhance the performance of GI. More specifically, the goal of our technique is to reconstruct the target image quickly and accurately at low sampling rate. A novel deep learning ghost imaging (DLGI) method is proposed that is consistent with the GI principle. Although the CS algorithm has a large computational challenge to acquire an ideal target image, it can reconstruct a rudimentary target image quickly at low sampling rate. Therefore, the CS algorithm is utilized in the preprocessing procedure to obtain a rudimentary target image quickly as the input of the network. Within the broad field of deep learning, the convolutional neural networks (CNN) are widely used for image recognition^38–40^. Mousavi *et al*. proposed a fast reconstruction algorithm based on deep CNN network, which can reconstruct the original image with fewer sample points. The proposed method solves the problem of low efficiency and limited application conditions of CS algorithm^41^. Usually, the CNN output is a scalar that is defined as a classifier. Our goal is to complete the image reconstruction. Consequently, the input and output of conventional CNN are modified to fit our needs. The new CNN used in our DLGI method is called GI convolutional neural network (GICNN). By utilizing GICNN, input data at low sampling rate can be trained. As a result, a complete mapping can be built between the input data and the label. The target image can be quickly and accurately reconstructed at low sampling rate, when testing. The schematic diagram of the DLGI method is shown in Fig. 1.

In this paper, a novel deep learning ghost imaging method is proposed, which has advantages both in calculation speed and image accuracy. A special convolutional neural network is designed according to the features of GI. Our method can also be used for other conventional imaging methods using similar imaging processes to GI.

## Results

**Figure 2:**
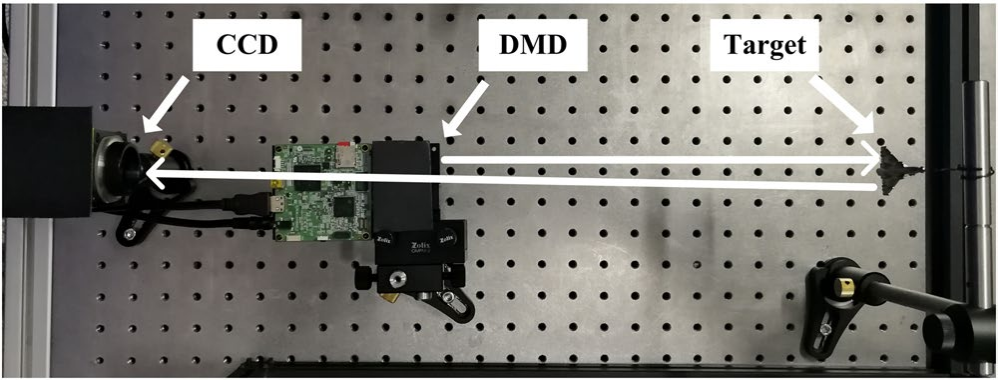
Schematic of the Experiment. The target is an aircraft model. The DMD is placed slightly lower to avoid blocking the echo signal. The photon flux was about 30 *μ*W/cm^2^. The distance between aircraft model and DMD is 27.5 cm. From aircraft model to CCD is 45 cm, and from CCD to DMD is 17.5 cm.

Our imaging configuration is based on the CGI framework^8^, as shown in Fig. 2, which consists of a target (an aircraft model), a Digital Micro-mirror Device (DMD), and a bucket detector. We used a CCD camera to simulate the bucket detector by summing all the pixels to obtain the whole intensity. The DMD projects a sequence of 64*64 random speckles onto the target, and the reflected light is detected by the CCD that gives the echo signals. Here, we define the full sampling rate as 4096 (64*64) detections. Correspondingly, 20% sampling rate stands for 819 detections.

### Simulation Testing

**Figure 3:**
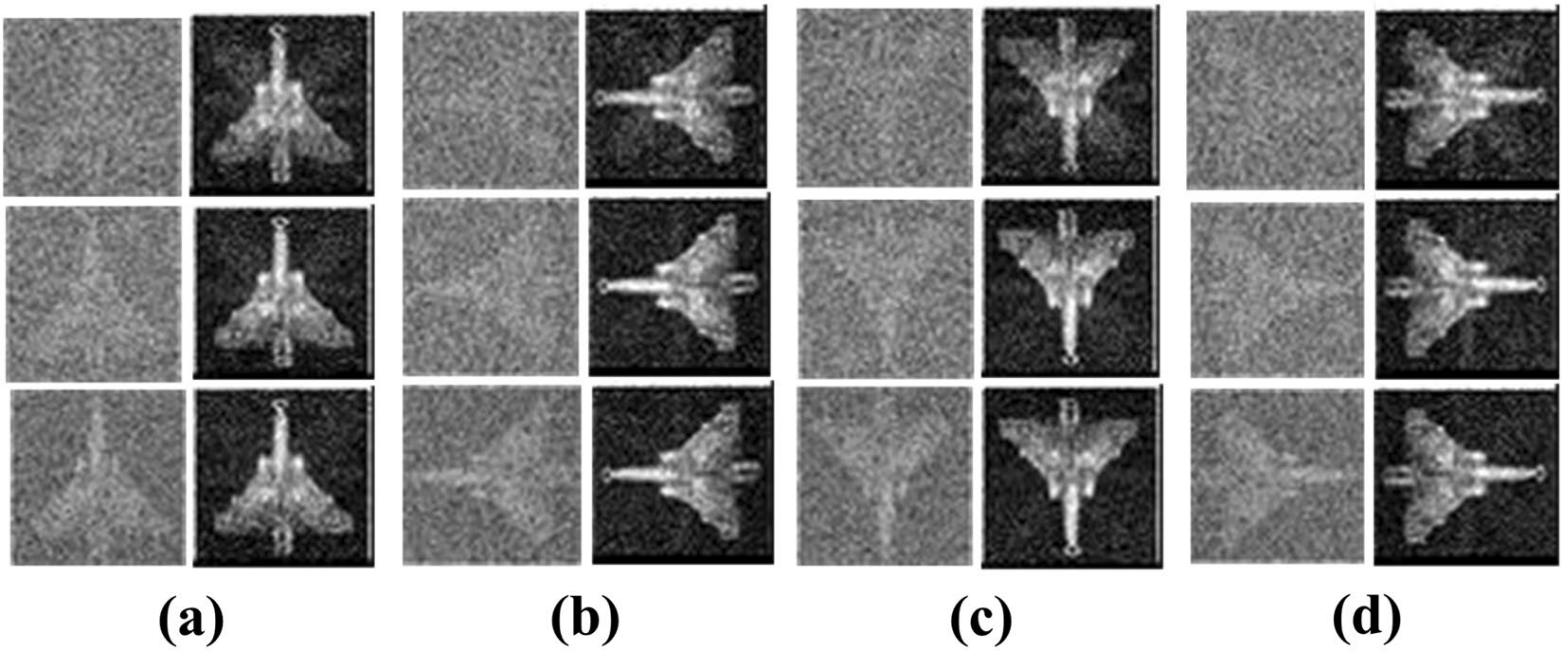
Simulation Results. (**a**–**d**) Show the four different attitudes, respectively. The first line is 5% sampling rate. The second line is 10% sampling rate. The third line is 20% sampling rate.

Firstly, we carried out numeriacal simulation for the whole imaging process based on the setup of Fig. 2. In order to train the GICNN, we randomly selected 100 samples with 20% sampling rate as training samples. Every sample has a label with full sampling rate. After training, 10 other samples were entered for testing. Three different sampling rates (5%, 10% and 20%) were selected to test the effect of DLGI method. The simulation testing results are shown in Fig. 3.

Independent of the sampling rate, the system is capable of obtaining the target image from a rudimentary input. With such low sampling rates, both the basic correlation method and the CS algorithm cannot acquire a clear target image quickly. The image qualities improve with increasing sampling rates. Furthermore, we changed the attitude of the target for training and testing to better demonstrate the GICNN. The head of aircraft is rotated 90 degrees, 180 degrees and 270 degrees, respectively. It can be seen from Fig. 3(a–d) that we can obtain the target image from the rudimentary input regardless of attitude. The simulation was conducted to verify the method. To better demonstrate our method, a real scene physical experiment is carried out below.

### Physical Experiment

**Figure 4:**
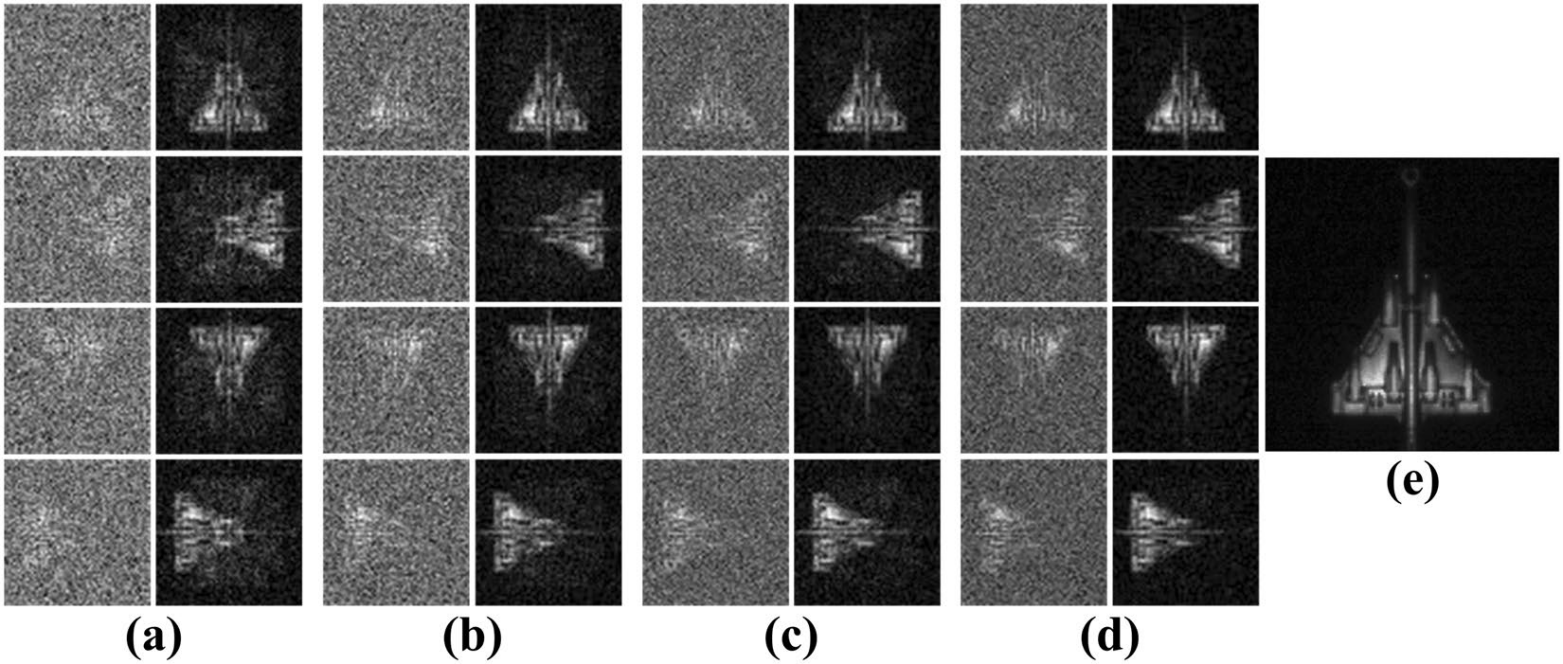
Experimental Results. (**a**–**d**) Show the results of the aircraft model at different attitudes using 5%, 10%, 15% and 20% sampling rates, respectively. (**e**) Is the training label obtained by the CCD camera.

The schematic of the physical experiment is shown in Fig. 2. We performed 300000 bucket detections to form sample set. Subsequently, 200 samples were randomly selected from the sample set for training, and 100 other samples were randomly selected from sample set for testing. Similarly, the GICNN was trained at 20% sampling rates. The 5% sampling rates, 10% sampling rates, 15% sampling rates and 20% sampling rates were tested. The experimental results are shown in Fig. 4.

Figure 4 shows that a clear target image can be obtained after the testing data had passed through the network. As the sampling rate increases, the image becomes clearer. When the sampling rate reaches 20%, the target image can be practically reconstructed. We compared DLGI with other conventional GI algorithms with respect to peak signal-to-noise-ratio (PSNR) and imaging time. Note that, data collection time was not considered in our comparison, the results are the pure time consuming of reconstruction.

**Figure 5:**
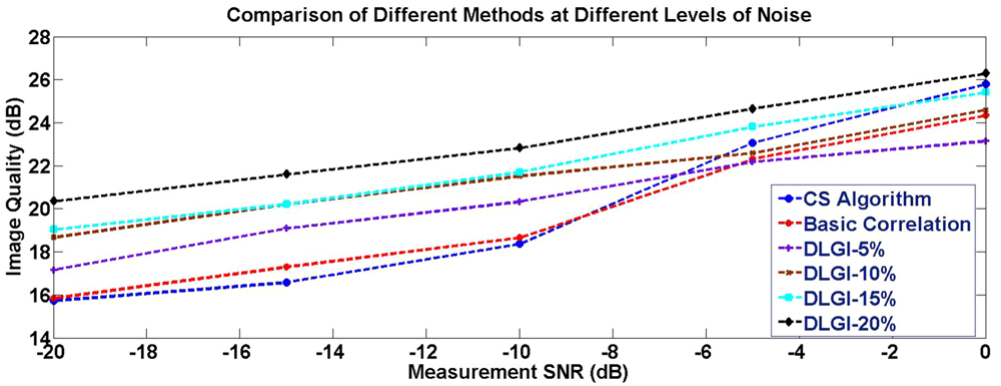
Results of the Comparison. The CS algorithm, basic correlation method and DLGI method with four different sampling rates (5%, 10%, 15% and 20%) were selected in our comparison experiments.

Table 1 shows that the DLGI method shortens the imaging time considerably. When the PSNR of the output images are similar (about 26 dB, just like the image quality in Fig. 4(d)), the DLGI method at 20% sampling rate is much faster than the basic correlation method and the CS algorithm. In our physical experiment, the sparsity of the aircraft scene is low. Therefore, the PSNR of CS result is not ideal with under-sampling. To compare with other methods under similar PSNR, the sampling rate for CS was also full sampling rate. As mentioned at the beginning of the results section, the full sampling means 4096 (64*64) detections. Note that, the results of DLGI method at other sampling rates are also much faster than both the basic correlation method and the CS algorithm. As the sampling rate increases, the imaging time decreases. The time of transforming the echo signal into a rudimentary image was not been considered, which was less than 1 second. Despite the transformation time, the results of DLGI method are still much faster than other methods. In addition, we added different levels of noise during the data acquisition process in our experiment. In particular, we selected 0 dB, -5 dB, -10 dB, -15 dB and -20 dB for a comparison. The image qualities were evaluated based on PSNR. The results of the comparison are shown in Fig. 5.

**Table 1:** Comparison Results.

Method	PSNR (dB)	Time (s)
5% sampling rate DLGI	23.219	0.0036
10% sampling rate DLGI	24.016	0.0032
15% sampling rate DLGI	25.337	0.0031
20% sampling rate DLGI	26.303	0.0023
Basic Correlation Method	26.620	0.31
Compressed Sensing Algorithm	26.521	5.8

Figure 5 shows that the image qualities improve with the increasing of the measurement SNR. However, the image qualities of the DLGI method are significantly better than the other methods under all levels of the measurement SNR. The best performance is the DLGI method at 20% sampling rate. Even though the image qualities of the CS algorithm and basic correlation method do not differ a lot from the DLGI method, their required processing-times are much longer than the DLGI method - see Table 1.

### Diverse Scenes

**Figure 6:**
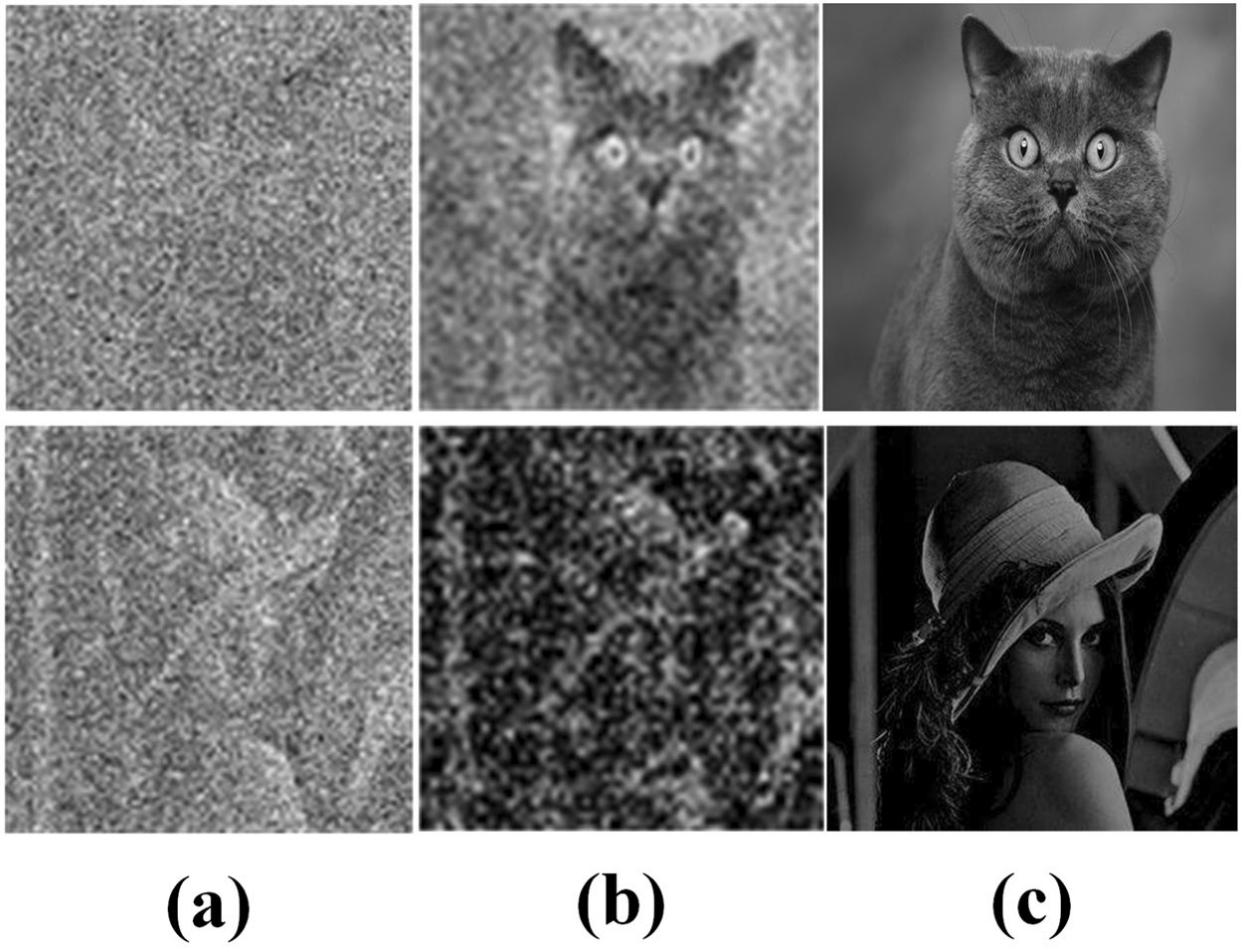
Results of Diverse Scenes. (**a**–**c**) Show the input, the reconstructed result and the target, respectively.

In order to study the applicability of our method in different complexities scenes, we carried out different nature scenes including the cat and Lena. The results are shown in Fig. 6.

The amount of training data were all 500 samples for the two scenes. The PSNR of the cat in Fig. 6(b) is 16.3713 dB, and Lena is 15.1714 dB. The results show that the simple scenes (the cat) can obtain better results under same amount of training data. Then, we increased the training data of complex scenes (Lena).

**Figure 7:**
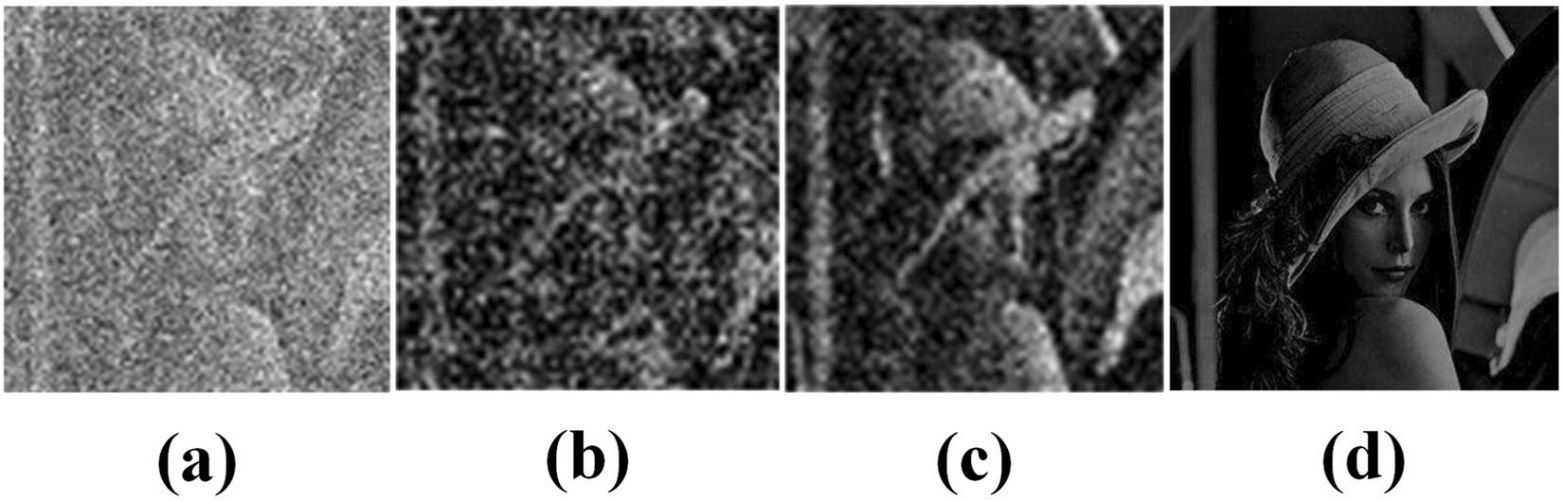
Results of Increasing the Amount of Training Data. (**a**–**d**) Show the input, the reconstructed result under 500 samples, the reconstructed result under 1000 samples and the target, respectively.

We increased the amount of training data of Lena from 500 samples to 1000 samples. The PSNR of Lena in Fig. 7(c) is 16.0147 dB. Figure 7 shows that with the increase in the amount of training data, the reconstruction results of complex scenes are enhanced. The above results indicate that different complexities scenes require different amount of training data. The complex scenes require large amount of training data, and the simple scenes require small.

## Discussion

**Figure 8:**
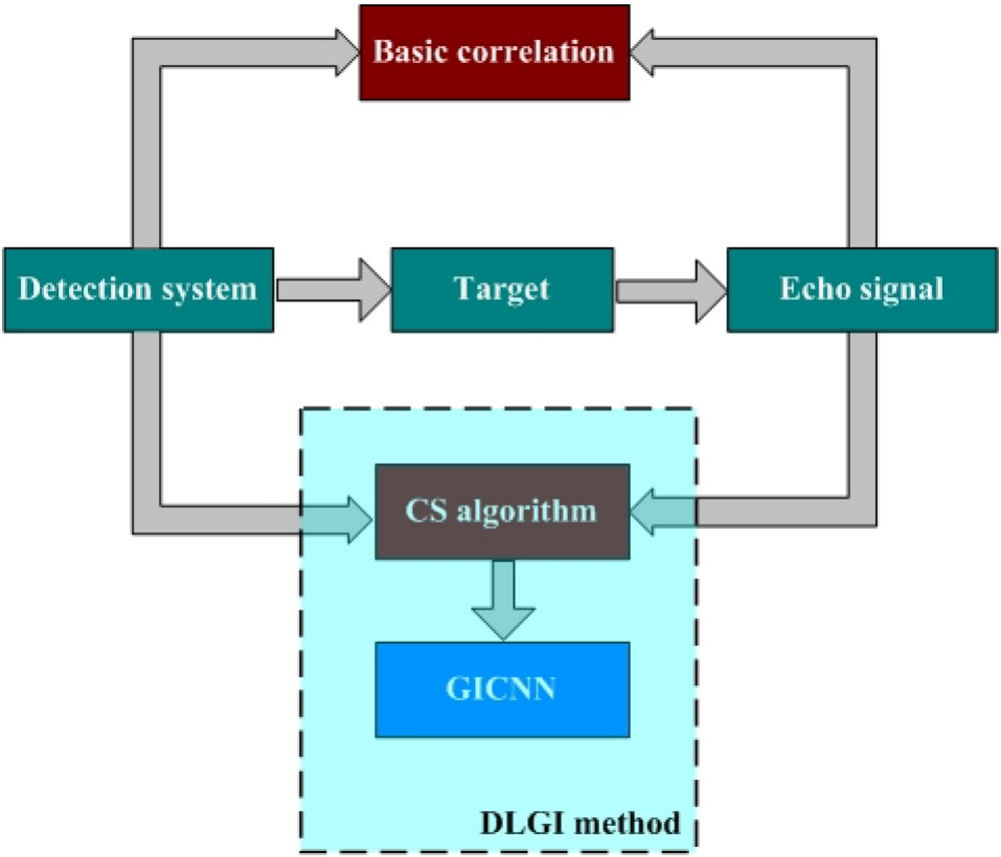
Different Processing Methods of GI. The current mainstream ghost imaging processing methods are given.

Our study presents a novel deep learning GI method. The optimization through artificial intelligence has been successfully used in many other fields, we investigated this possibility in this paper. GI utilizes the correlation between reference arm and signal arm to complete the image reconstruction of the target^1^. CGI uses calculation instead of detection to obtain the information in the reference arm^8^. In other words, GI is an imaging method, where a target is reconstructed from the received signal if the detection signal is known and random. Figure 8 shows the different methods involved in GI.

Performing the correlation calculation between echo signal and the independent speckle patterns at target plane directly is the basic correlation method.

1 $${g}^{(2)}=\frac{ < {I}_{b}\cdot I(x,y) > }{ < {I}_{b} >  < I(x,y) > }\,$$

where 〈·〉 means the ensemble average. *I*_*b*_ represents the echo signal that the bucket detector received and I(*x*, *y*) represents the speckle patterns distribution in the target plane. Subsequently, the target image can be obtained through normalized correlation calculation between them. Although this method is simple, the effective is low. The CS algorithm we employed is fast iterative shrinkage threshold algorithm (FISTA)^42,43^. FISTA is suitable for the preprocessing procedure reconstruction because it converges quickly. The reconstruction model, based on FISTA, can be summarized as follow

2 $$\hat{X}={\rm{\arg }}\,\mathop{{\rm{\min }}}\limits_{X}\{\frac{1}{2}\parallel Y-AX{\parallel }_{2}^{2}+\lambda \parallel \alpha {\parallel }_{1}\},$$

where *α* = Ψ · *X* denotes the sparse in the representation basis Ψ, *λ* is regularization parameter. *y* represents the data received by the bucket detector, *A* represents the speckle patterns at the target plane, and *X* represents the target to be reconstructed.

All methods require the independent speckle patterns at target plane as well as an echo signal, but the method we proposed is different from the basic correlation method and the CS algorithm. Our approach does not change the data acquisition procedure of conventional GI method, it opens doors to improvement using modern deep learning principles. Optimization using the model in Eq. (2) can quickly produce a rudimentary target image for CNN. CNN utilizes several layers like convolution layer, pooling layer and linear layer to extract the characteristics of the input image. By constantly training and adjusting the parameters within the network, CNN can perform complete mapping between input and output. In the convolution layer, a weight matrix was utilized to extract the feature information from the input image, which can be regarded as a filter. The filter moves on the picture at a certain step size. A combination of different weight is used to extract a feature class. Another combination of weight is used to extract another feature type. It is necessary to learn the parameters from the input image to extract information so that the network can perform a correct prediction. As the network structure becomes deeper, the characteristics of weight matrix extraction become more complex and increasingly applicable to solve the given problem. The convolution parameters affect the result greatly. Sometimes the image is too large and we need to reduce the number of training parameters. It is necessary to introduce the pooling layer periodically between the convolution layers to reduce the image size. The ramp function is chosen as the activation function. Normally, a conventional linear layer outputs various classes to produce a one-dimensional vector to output the most likely of them. We redesigned the the output of the linear layer to obtain the target image. From the above simulations and experiments we can see that the DLGI method we proposed is effective, and deep learning can be used successfully in GI. The target can be reconstructed by our method should be in the data set that we have trained. The more kinds of targets are trained, the more targets can be reconstructed. Meanwhile, the applicability of our method for an unknown target imaging will be the focus of our next step.

In conclusion, a novel deep learning GI method is proposed. We modify the convolution neural network according to the features of GI. Then, a clear target image can be obtained when the under sampled data pass through our trained network. A series of simulations and experiments results show that the DLGI method can obtain the target image faster and more accurate at low sampling rate compared with conventional GI method. Our method provides a way of introducing the artificial intelligence into GI. Moreover, other imaging methods similar to GI process can be applied to our method.

## Methods

**Figure 9:**
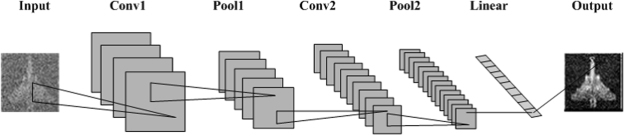
Schematic of the Network. Our network consists of two convolution layers, two pooling layers and one linear layer. The ramp function was added to serve as activation function between the first convolutional layer and the pooling layer, between the second convolutional layer and the pooling layer, as well as between the second pooling layer and the last linear layer.

In this section, a detailed introduction to the network in DLGI method is presented. Because the CNN requires a picture as the input, a preprocessing procedure is added. The purpose of the preprocessing procedure is to complete the initial image reconstruction so that the rudimentary target image can be offered to CNN. The CS algorithm is utilized to obtain the rudimentary target image. Convolution layer, pooling layer and linear layer are selected to build our network. We added the ramp function between conv1 and pool1, conv2 and pool2, pool2 and linear to eliminate the negative number. Note that, the output of the linear layer was redesigned to produce the target image. The linear layer contains each pixel of the target image. Each pixel has its own weight, and the weight parameters are continually adjusted according to the input during the training process. Depending on the size of the desired output image, we reshaped this one-dimensional vector into the output target image. The number of pixels in the final output target image is equal to that in the one-dimensional vector. The normalized mean squared error (NMSE) is used as a loss function to adjust the difference between the output and the label. We used Mathematica 11.1 and a desktop computer with an NVIDIA Quadro K4000 graphics card to train our network. The network is shown in Fig. 9.

## Electronic supplementary material

Dataset 1
